# Road User Position and Speed Estimation via Deep Learning from Calibrated Fisheye Videos

**DOI:** 10.3390/s23052637

**Published:** 2023-02-27

**Authors:** Yves Berviller, Masoomeh Shireen Ansarnia, Etienne Tisserand, Patrick Schweitzer, Alain Tremeau

**Affiliations:** 1 Institut Jean Lamour, Université de Lorraine, UMR7198, F-54052 Nancy, France; 2 Independent Researcher, F-57155 Marly, France

**Keywords:** ADAS, I2V, deep learning, camera to world transform, real time

## Abstract

In this paper, we present a deep learning processing flow aimed at Advanced Driving Assistance Systems (ADASs) for urban road users. We use a fine analysis of the optical setup of a fisheye camera and present a detailed procedure to obtain Global Navigation Satellite System (GNSS) coordinates along with the speed of the moving objects. The camera to world transform incorporates the lens distortion function. YOLOv4, re-trained with ortho-photographic fisheye images, provides road user detection. All the information extracted from the image by our system represents a small payload and can easily be broadcast to the road users. The results show that our system is able to properly classify and localize the detected objects in real time, even in low-light-illumination conditions. For an effective observation area of 20 m × 50 m, the error of the localization is in the order of one meter. Although an estimation of the velocities of the detected objects is carried out by offline processing with the FlowNet2 algorithm, the accuracy is quite good, with an error below one meter per second for urban speed range (0 to 15 m/s). Moreover, the almost ortho-photographic configuration of the imaging system ensures that the anonymity of all street users is guaranteed.

## 1. Introduction and Related Works

Self-driving vehicles or autonomous road vehicles have constituted a very active field of research in recent years among Intelligent Transportation Systems, which covers many aspects of transportation. One task in autonomous driving is perception and is often based on the use of Light Detection and Ranging (LiDAR) [1,2] or on the use of visual recognition systems (VRSs) [3], or a fusion of both [4,5]. VRS encompasses image classification, object detection, segmentation, and localization [6]. In most cases, image processing is performed using cameras mounted on a vehicle [7] and focuses on pedestrian detection [8]. The coexistence of autonomous vehicles with vehicles driven by humans is also an issue that has been raised [9]. The vehicle fleet share of automated vehicles started to significantly increase from 2020 onward but may still be less than 25% of all vehicles in 2030 [10]. It is, therefore, interesting to provide useful information to motorists using both standard vehicles and autonomous vehicles. In the presented work, we address, more specifically, urban road users through Advanced Driving Assistance Systems (ADASs). We use the Infrastructure to Vehicle (I2V) communication system to significantly improve safety via the transmission of a small amount of high-level data extracted from camera images, thanks to the use of deep learning algorithms. Our work is devoted to omnidirectional trajectories of the detected objects and can deal with multiple travel modes (automotive, motorcycle, bicycle, pedestrian, and so on). It is based on a fully passive method (no RADAR or LIDAR required) and the only required sensor is a camera. We make full use of artificial neural networks for the detection (and optionally for the segmentation) and the motion estimation of the objects of interest. We also take into account the quasi ortho-photographic setup of the camera in order to simplify the camera to world coordinates transformation. This kind of bird’s-eye view also minimizes the risk of object occlusions compared to a traditional perspective view. The work presented in this paper is quite similar but complementary to that of [11], since the latter deals only with the detection of pedestrians, whereas we deal with all road users.

The main contributions of our work are:

- The use of a fine analysis in the optical setup of the camera;
- The presentation of a detailed procedure to obtain the Global Navigation Satellite System (GNSS) coordinates in real-time;
- The speeds of the moving objects, although being computed offline for now;
- All the information extracted from the image by our system represents a small payload and can easily be broadcast to road users;
- The transmission of the GNSS coordinates (latitude *φ*, longitude *ω*, and label) of each detected object *u* ensures that the navigation applications for final users can identify themselves and filter out non-pertinent data;
- Since the system resides in the infrastructure and can benefit all road users, it naturally optimizes embedded computing resources and energy consumption.

Finally, we partly address edge computing and we do not deal with the transmission delay added by the network communication. The latter is well dealt with by [11].

**Figure 1:**
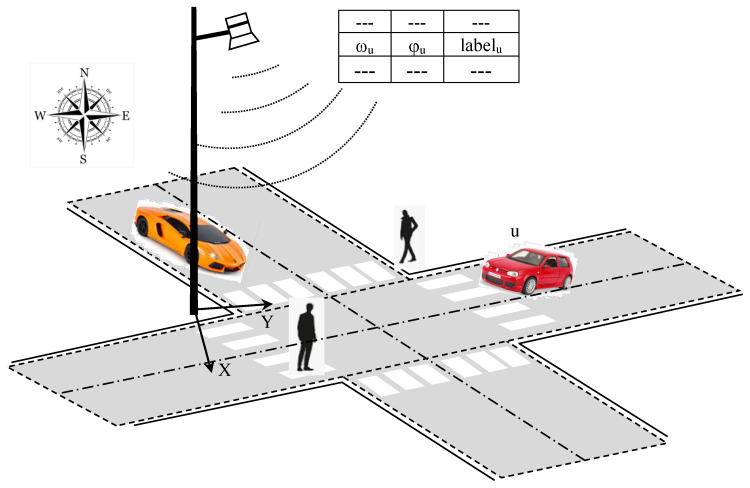
I2C communication between supervisor system and vehicles.

The final goal of this study is to warn road users about potential collisions. Figure 1 describes the principal scheme of the final system.

The remainder of the paper is organized as follows. In Section 2, we present all the setup and processing required for computing the camera to world coordinate transform. Section 3 enumerates the deep-learning-based algorithms, which provide the labels, locations, and velocities of the detected users in the scene relative to the camera position. Section 4 describes the processing that leads to the determination of the GNSS coordinates of each detected road user ready for the transmission over I2V communication and presents some results. Finally, in Section 5, we discuss some limitations, and Section 6 concludes with a list of future works.

## 2. Optical Setup

### 2.1. Shooting Configuration

The field of view of a camera derives directly from the geometric configuration of the shot (position and angles) and the characteristics of the optical system. Knowing that, in practice, it will not be possible to change these parameters, their choice is crucial.

**Figure 2:**
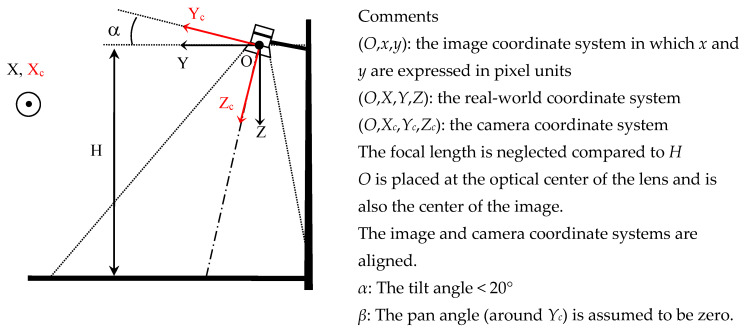
Configuration and shooting coordinate systems.

The camera is attached at a height *H* above the ground. The multitude of use cases and user behaviors requires an observation area in front and behind the mast, which leads to directing the camera towards the ground along an almost vertical axis, as shown in Figure 2.

**Figure 3:**
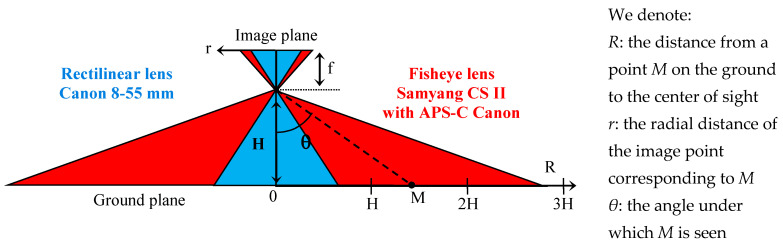
Compared field of view of two lenses.

This quasi ortho-photographic configuration offers several advantages:

- The FoV and the height *H* of the camera are the only geometrical parameters of the installation;
- The area of interest is centered on the optical axis. For standard optics, the sky is absent from the images, limiting the risk of solar glare;
- The quasi ortho-photographic configuration allows for easier horizontal plane segmentation and ground distance estimation;
- The location of an object in the horizontal plane is precise and simple to determine;
- The structures, such as advertising poles, lamp posts, and red lights, which are very present in urban areas, are well-suited supports for attaching the camera;
- Detection humans in this perspective could not be subject to mass surveillance since facial features are practically impossible to recognize;
- The risk of moving object occlusions is significantly reduced.
- On the other hand, several drawbacks can be noted:
- As shown in Figure 3, a traditional rectilinear optical projection system provides limited range. Fisheye optics makes it possible to remedy this problem at the cost of a strong radial distortion of the images;
- Deep learning algorithms were mainly developed and trained on frontal shots. Their use in this context requires specific training.

### 2.2. Lens Selection

All our experiments are carried out with a Canon APS-C EF mount sensor fitted with a Samyang CS II lens.

**Figure 4:**
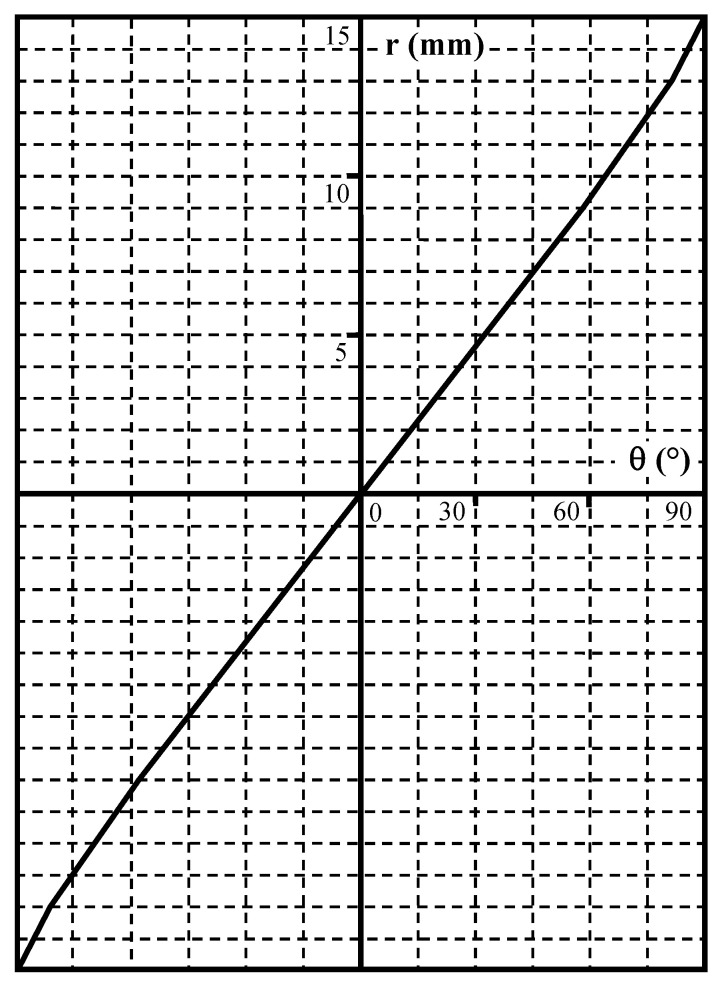
Radial mapping of Samyang CS II lens.

The characteristic *r*(*θ*) recorded for this lens with a 24 × 36 mm retina is represented in Figure 4. It has an acceptable linearity of 0.16 mm/° in the angular range extending from −75° to +75°.

This linearity corresponds to an equidistant optical projection represented by Equation (1)

(1) r=f·θ

where *f* is the slope expressed in mm/rad or pixel/rad.

Compared to the APS sensor (22.3 × 14.9 mm), which equips our reflex (Canon EOS 2000D), we obtain an FoV in panorama mode of 139° according to the width, 93° according to the height, and 167° according to the diagonal.

These angular values provide us with an observation surface on the ground equal to approximately 5.5*H* × 2.1*H* in perfectly ortho-photographic mode.

### 2.3. CamToWorld Transform

We use the equidistant projection model to work out the inverse transformation, which makes it possible to determine the *X* and *Y* positions of a point *M* on the ground from the coordinates (*x*, *y*) of its image.

Then, for radial positions, we have:

(2) $$R=\sqrt{X^{2}+Y^{2}}=Htg\left(\theta \right)$$

(3) $$r=\sqrt{x^{2}+y^{2}}$$

For the projection model:

(4) r=f·θ

(5) $$\frac{Y}{X}=\frac{y}{x}$$

Substituting (4) and (5) into (2), we obtain:

$$=X\sqrt{1+\frac{Y^{2}}{X^{2}}}=X\sqrt{1+\frac{y^{2}}{x^{2}}}=\frac{X}{x}r=\frac{Y}{y}r=H\cdot tg\left(\frac{r}{f}\right)$$

By setting $F\left(r\right)=\frac{1}{r}\cdot tg\left(\frac{r}{f}\right)$, the distortion function introduced by the lens, we obtain the transformation Equations (6) and (7)

(6) $$X=H\cdot x\cdot F\left(r\right)$$

(7) $$Y=H\cdot y\cdot F\left(r\right)$$

Now, taking into account the tilt angle *α*:

The coordinates of a point on the ground in the scene coordinate system are (*X*, *Y*, *H*). In the camera coordinate system, they become:

(8) $$\left(\begin{array}{c} X_{c}\\ Y_{c}\\ Z_{c} \end{array}\right)=\left[\begin{array}{ccc} 1 & 0 & 0\\ 0 & cos\left(\alpha \right) & sin\left(\alpha \right)\\ 0 & -sin\left(\alpha \right) & cos\left(\alpha \right) \end{array}\right]\cdot \left(\begin{array}{c} X\\ Y\\ H \end{array}\right)$$

In Equations (6) and (7), we have to replace *X*, *Y*, and *H*, respectively, with *X_c_*, *Y_c_*, and *Z_c_* and then replace the latter by their relations with *X*, *Y*, and *Z* given by (7).

These operations make it possible to obtain the linear system (9), which makes it possible, by inversion, to determine the real-world *X* and *Y* coordinates of the points of the ground from the pixel coordinates.

(9) $$\left[\begin{array}{cc} 1 & x\cdot F\cdot sin\left(\alpha \right)\\ 0 & cos\left(\alpha \right)+y\cdot F\cdot sin\left(\alpha \right) \end{array}\right]\left(\begin{array}{c} X\\ Y \end{array}\right)=H\cdot \left(\begin{array}{c} x\cdot F\cdot cos\left(\alpha \right)\\ y\cdot F\cdot cos\left(\alpha \right)-sin\left(\alpha \right) \end{array}\right)$$

Finally, we obtain the Camera To World transformation Equations (10) and (11):

(10) $$X=\frac{H}{K}\cdot x\cdot F$$

(11) $$Y=\frac{H}{K}\cdot \left(y\cdot F\cdot cos\left(\alpha \right)-sin\left(\alpha \right)\right)$$

$$\mathrm{With}\;K=cos\left(\alpha \right)+y\cdot F\cdot sin\left(\alpha \right)\;\mathrm{and}\;F=\frac{1}{r}*tan\left(\frac{r}{f}\right)$$

For another type of fisheye lens, the projection function *r* = *func*(*θ*) is taken into account in the CamToWorld transformation in the form of the distortion function $F\left(r\right)=\frac{1}{r}\cdot tg\left(func^{-1}\left(r\right)\right)$.

### 2.4. Experimental Verification

**Figure 5:**
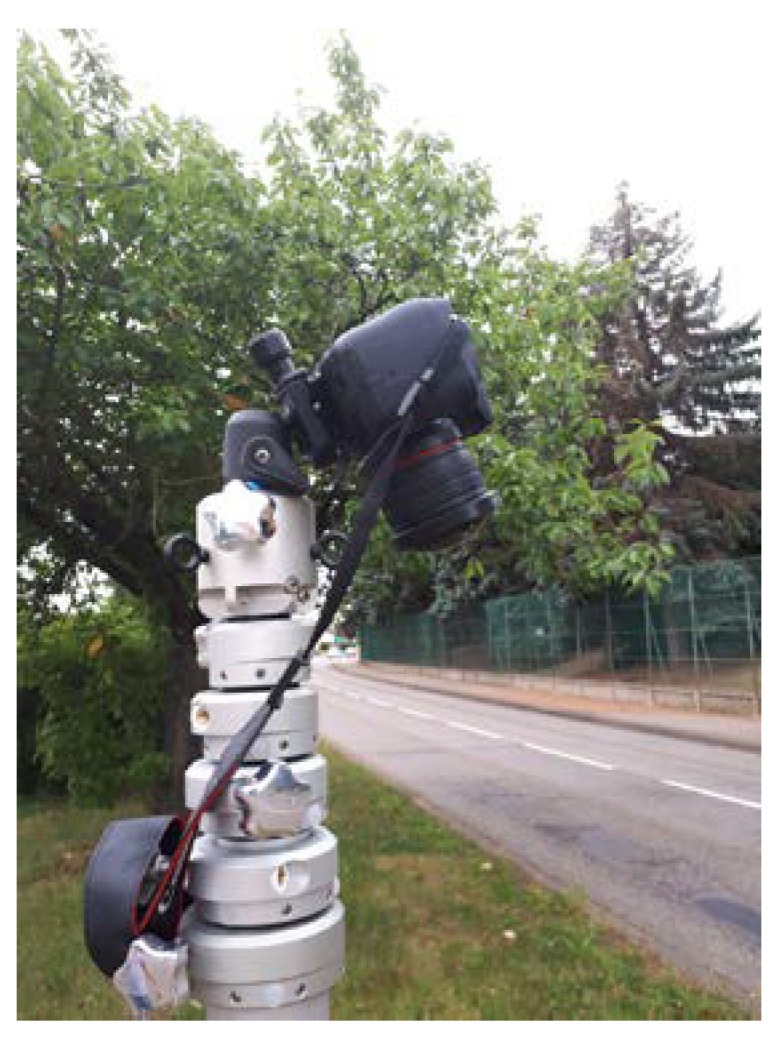
Image recording system.

We checked the ability to estimate the actual positions and dimensions of road elements using the camera system. The reflex camera is fixed on a telescopic mast whose height *H* can vary between 2 and 8 m (Figure 5). For images of 1920 × 1080 pixels, the parameter *f* is established at 789.3 pixel/rad.

**Figure 6:**
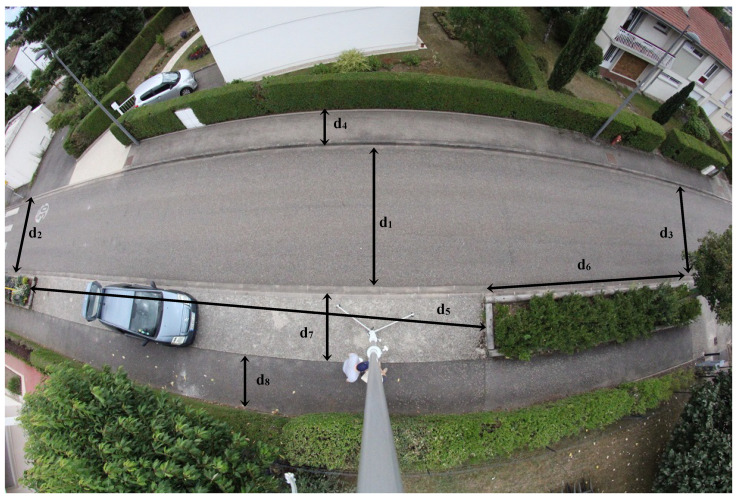
Fisheye view of the area with *H* = 7 m.

**Figure 7:**
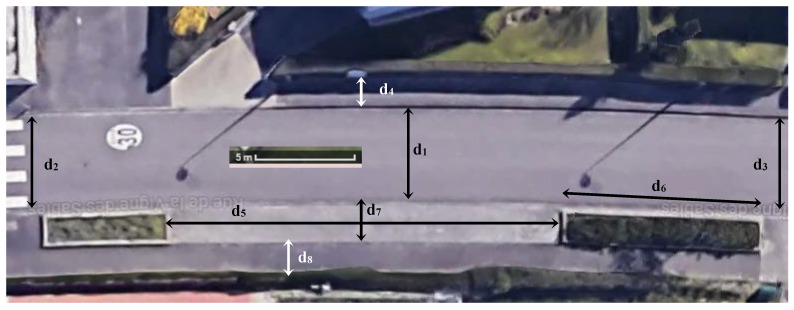
Aerial photograph of the same area.

We present, here, the results obtained on the snapshot (Figure 6) taken at the point with GPS coordinates 48.659276, 6.195960, and *H* = 7 m. The aerial view of the area shown in Figure 7 allows the deformations to be assessed.

The different lengths on the ground are measured in the field (in the real world, on the ground). Table 1 allows us to assess the agreement of the estimates provided by the CameraToWorld transformation.

## 3. Image Processing Flow

### 3.1. Road User Detection

Ten years ago, the detection of road users and, particularly, pedestrians were mainly based on Histograms of Oriented Gradient (HOG) [12,13]. Nowadays, artificial neural networks and, more specifically, Convolutional Neural Networks are used for those tasks.

YOLO [14] has been widely used for real-time object detection in various applications and specifically for pedestrian detection [15] in video images. In our work, we use YOLOv4, implemented thanks to the TensorFlow library, and we tuned a COCO trained version with transfer learning to adapt it to near-ortho-photographic fisheye images. The YOLOv4 model was fine-tuned using a custom dataset of ortho-photographic fisheye images. The dataset was prepared by recording videos in a parking lot with a vertical angle of view and low illumination. Images were extracted from the footage, resulting in approximately 3000 images. Data augmentation techniques, such as rotation, flip, and brightness reduction, were applied to expand the dataset to approximately 8000 images. Training was carried out with a batch size of 16 and 500 epochs.

### 3.2. Optical Flow Analysis

In order to avoid the multi-object tracking task, which poses problems when the detection is incomplete, our speed estimation is based on the analysis of the intra-bounding box optical flow. This analysis can be performed on two successive frames of indexes *k* and *k* − 1 using Farnebäck’s analytical algorithm [16] or more quickly using a specialized CNN. In our system, this operation is carried out by the FlowNet2 CNN [17].

The highly probable hypothesis that we adopt assumes that the displacement of the same object between two successive images does not exceed half the dimension of its bounding box so that the analysis covers more than half of the mobile.

For a 4 m-long vehicle and a video rate of 20 FPS, this assumption is no longer verified for a speed greater than 40 m/s, a very rare situation in urban areas.

The analysis of the dense optical flow makes it possible to determine the apparent displacement in a video sequence for each pixel in the image.

The optical flow can be exploited in our study to evaluate the vector $\vec{d}=\left(u_{m},v_{m}\right)$ of the average apparent displacement in each detected bounding box.

**Figure 8:**
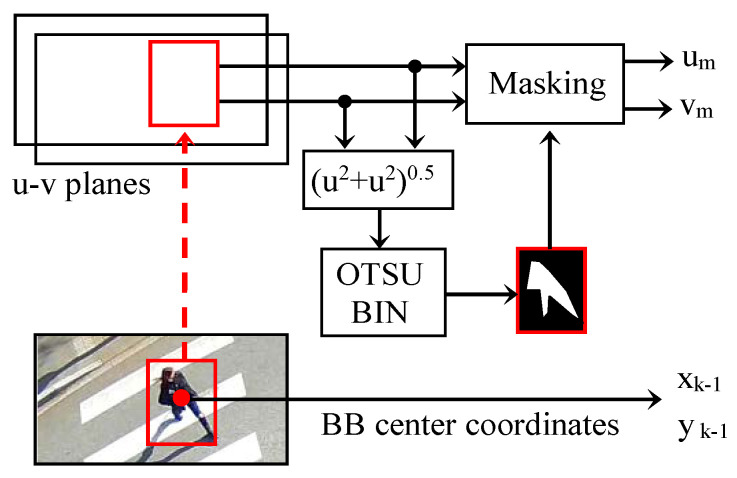
Calculation procedure of the average optical flow in a bounding box.

We assume that in each bounding box, the optical flow histogram is bimodal. It comprises a first set of values (*u*, *v*) in which the movements of small amplitude and of any orientation are grouped. These are noises or movements independent of that of the object concerned. The second set is characterized by larger and more oriented movements because they are strongly correlated to the overall displacement of the object. The separation of these two classes can, thus, be performed efficiently using Otsu’s automatic binarization procedure. The calculation of the average displacement vector $\vec{d}$ is then carried out only on the pixels selected by the binarization. A diagram for the evaluation of the displacement vector of a bounding box is represented in Figure 8. The procedure is repeated for each bounding box detected by YOLO.

The coordinates (*x_k_*, *y_k_*) in the center of the bounding box in image *k* can be approximated by:

$$x_{k}=x_{k-1}+u_{m}\;\mathrm{and}\;y_{k}=y_{k-1}+v_{m}$$

The coordinates *X_k_*, *X_k_*_−1_, *Y_k_*, and *Y_k_*_−1_ are determined by introducing, respectively, the values *x_k_*, *x_k_*_−1_, *y_k_*, and *y_k_*_−1_ in the pair of Equations (11) and (12).

**Figure 9:**
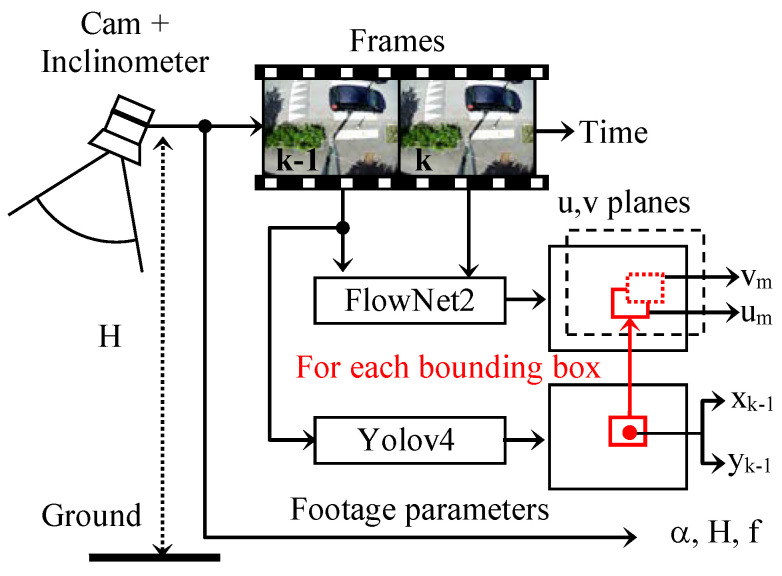
Optical flow determination.

**Figure 10:**
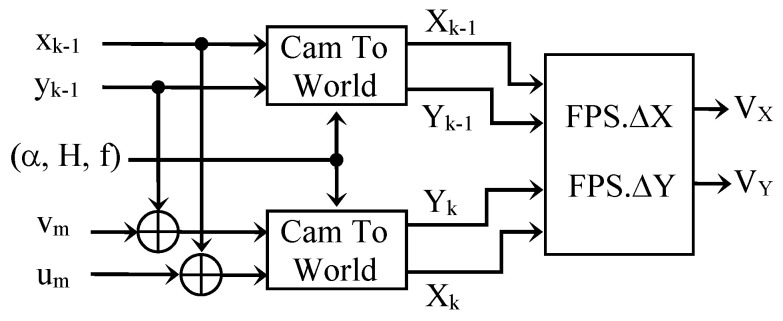
Actual speed estimation by optical flow determination.

The components *V_X_* and *V_Y_* of the actual speed vector are then obtained by multiplying Δ*X* and Δ*Y* by the video rate FPS. The diagrams given in Figure 9 and Figure 10 summarize the adopted procedure.

### 3.3. Detection and Actual Speed Estimation Results

In order to validate the operation of the system, we first made videos on a private site at night. In these experiments, the speed of the test vehicle was stabilized and its value recorded directly from the speedometer.

Yolov4 detection is performed in real time on RTX 4000 GPU. The *u* and *v* planes delivered by FlowNet2 (.flo files) were calculated offline on Google Colaboratory. By offline, we mean that the video was previously recorded and then processed on Colab, in contrast with the real-time detection with a camera directly connected to the processing system.

**Figure 11:**
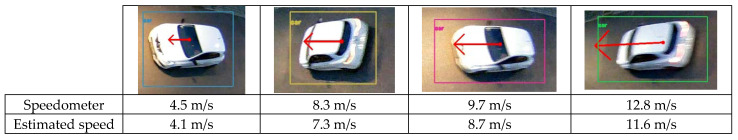
Estimation of speeds in private site and controlled conditions.

Figure 11 gives an overview of the detection and estimation results for speeds between 4 and 13 m/s. The speeds are recorded and estimated as the car passes the abscissa *X* = 0.

Knowing that speedometers slightly overestimate the measured speed for legal reasons, the estimates provided by the system seem consistent.

**Figure 12:**
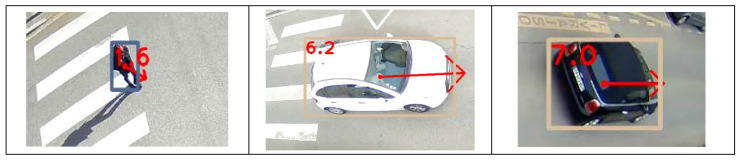
Estimation of the speed of anonymous users.

We then repeated the experiment on anonymous scenes shot on public roads. The photographs in Figure 12 give some examples. Velocity estimates in m/s are displayed on the images.

## 4. Data Extraction for I2V Communication

### 4.1. Transformation World to Vehicle’s Own Referential

The objective is to provide, in the reference frame of each vehicle, a dynamic horizontal map of nearby users. To carry out this cartography, knowledge of the position (*X_u_*, *Y_u_*) and velocity components *V_X_* and *V_Y_* of each vehicle in the ground plane (*O*, *X*, *Y*, *H*) is imperative.

*V_X_* and *V_Y_* are provided by the supervisor. The determination of *X_u_* and *Y_u_* requires knowledge of the GNSS (Global Navigation Satellite System) positions of the vehicle with respect to the position of the detecting camera.

The supervisor must transmit the positions (*X_u_*, *Y_u_*) of each detected mobile in GNSS format. This requires an additional transformation in the CameraToWorld, which is a rotation around the *Z* axis equal to the opposite of the azimuth (relative to geographic North) of the *Y* axis. This azimuth is obtained either by an electronic compass when installing the system or by an automatic calibration procedure based on knowledge of the GNSS coordinates of another (other than the base of the pole) point on the ground in the image. Once this rotation has been carried out, it remains to find the GNSS coordinates with reference to those of the foot of the post. In the general case, very precise and iterative methods make it possible to calculate the GNSS coordinates from a reference and an arbitrary displacement on the geodesic [18,19]. However, considering the small displacements in our application (at most, a few hundred meters) and the limited precision required (a meter), the approximation of the geodesic to a simple sphere of radius *R_earth_* is largely sufficient. Moreover, due to the former observations, the displacements along the latitude and the longitude can be processed independently (i.e., there is no need use spherical trigonometric equations).

**Figure 13:**
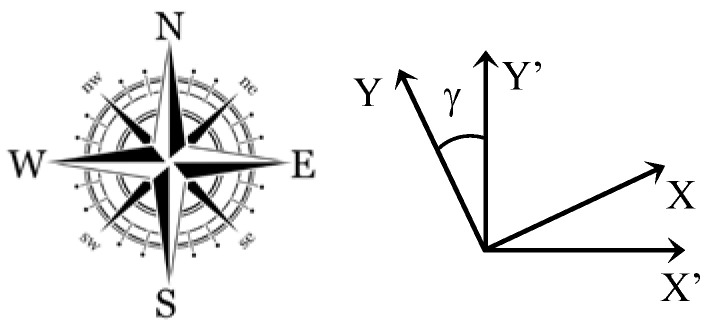
Metric coordinates in the WENS coordinate system.

Let (*X*’, *Y*’) be the metric coordinates in the WENS frame (Figure 13).

The conversion of the coordinates (*X*, *Y*) in the WENS frame of reference is obtained by an angle of rotation *γ*:

$$\left(\begin{array}{c} X\prime \\ Y\prime \end{array}\right)=\left[\begin{array}{cc} cos\gamma & sin\gamma \\ -sin\gamma & cos\gamma \end{array}\right]\cdot \left(\begin{array}{c} X\\ Y \end{array}\right)$$

where *γ* is the azimuth angle of the camera

### 4.2. Obtaining the GNSS Coordinates of Detected Users

The GNSS uses the spherical coordinates (*φ_M_*, *ω_M_*), where *φ_M_* and *ω_M_* are, respectively, the longitude and the latitude of the point M considered on the ground.

Knowing the GNSS position (*φc*, *ωc*) of the camera, the metric positions (*X’_c_*, *Y’_c_*) and (*X’_u_*, *Y’_u_*) of the camera and of the user in the WENS frame, we can determine the GNSS position (*φ_u_*, *ω_u_*) of this user.

**Figure 14:**
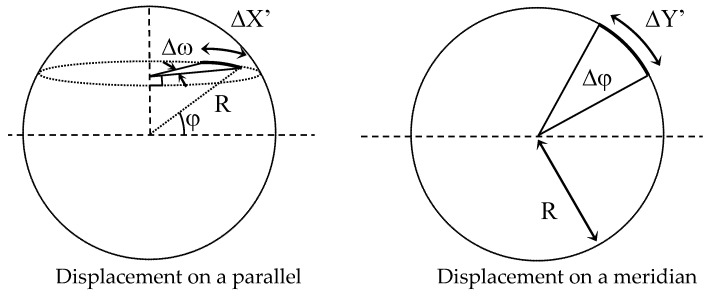
Displacement on a spheroid.

This determination calls upon the relations between displacements and angular variations on a meridian and a terrestrial parallel that, as shown in Figure 14.

We, thus, have:

$$\Delta X^{\prime }={X^{\prime }}_{u}-{X^{\prime }}_{c}=R_{earth}\cdot cos\left(\varphi \right)\cdot \Delta \omega \;\Delta Y^{\prime }={Y^{\prime }}_{u}-{Y^{\prime }}_{c}=R_{earth}\cdot \Delta \varphi$$

That is, for spherical coordinates:

$$\omega_{u}=\omega_{c}+\frac{\Delta X^{\prime }}{R_{earth}\cdot cos\left(\varphi \right)}\;\varphi_{u}=\varphi_{c}+\frac{\Delta Y^{\prime }}{R_{earth}}$$

In these relations, *R_earth_* is the terrestrial radius equal to 6,378,137 m, and the angles *ω* and *φ* are expressed in radians.

The display device (or a smartphone application) present in each nearby vehicle can, thus, superimpose the moving elements detected by the infrastructure on the representation used for navigation.

### 4.3. Detection and GNSS Localization Results in Night Conditions

A report drawn up in 2015 by the French road safety observatory shows that the majority of accidents involving a pedestrian occur during the day. Furthermore, 95% of these occur in urban areas. However, 37% of accidents causing the death of a pedestrian occur at night.

These statistics led us to verify the precision of the GNSS detection and localization of our system in low light conditions. To do this, we produced night video sequences, in which around twenty people walk through a parking lot on the university campus. The stage is illuminated by streetlights. It varies between 10 and 30 lux approximately. The height is fixed to *H* = 6 m. The exposure conditions used are: aperture *N* = 3.5, shutter time = 1/30 s, and ISO 1600.

**Figure 15:**
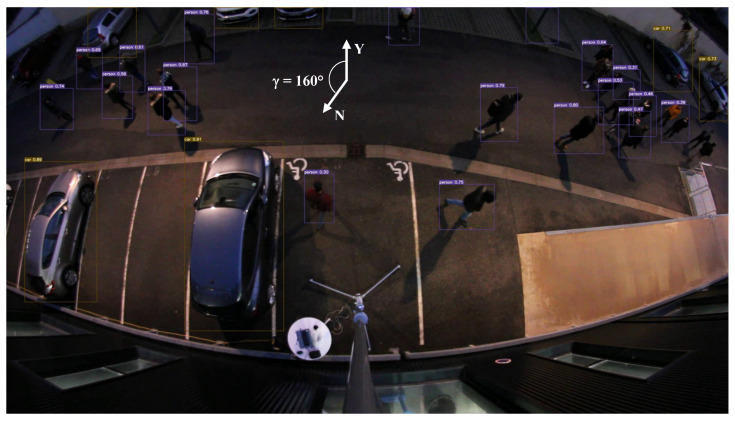
Detection of pedestrians and vehicles in night conditions.

The detection and GNSS localization are provided in real time at 15 FPS using a Jetson Nano GPU, in which Yolov4-Tiny, previously trained, was implemented. An image extracted from this video sequence is shown in Figure 15. The label (Person, Car), the latitude, and the longitude of the centers of the detected objects are saved in a spreadsheet file.

**Figure 16:**
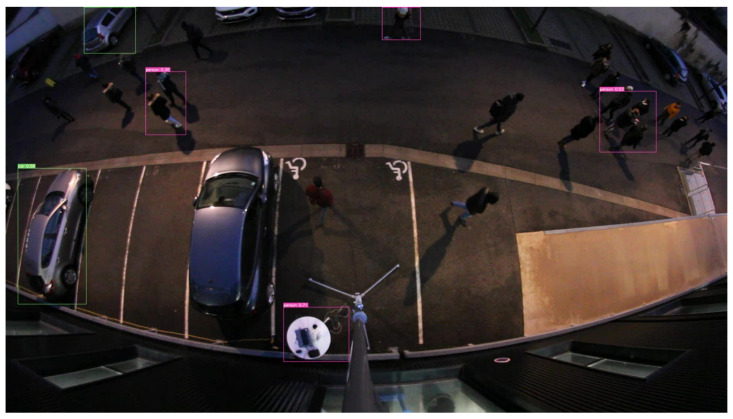
YOLOv7 trained on MS COCO only applied on the same image.

Since July 2022, YOLOv7 has constituted the state of the art in object detection. We tested this version trained on MS COCO only on our videos. Figure 16 gives an overview of the detection results. A comparison with Figure 15 shows the improvement brought by re-training our dataset of ortho-photographic fisheye images.

The positioning of detected users on a cadastral map can be easily achieved using the Python function library “plotly.express” (Figure 16).

**Figure 17:**
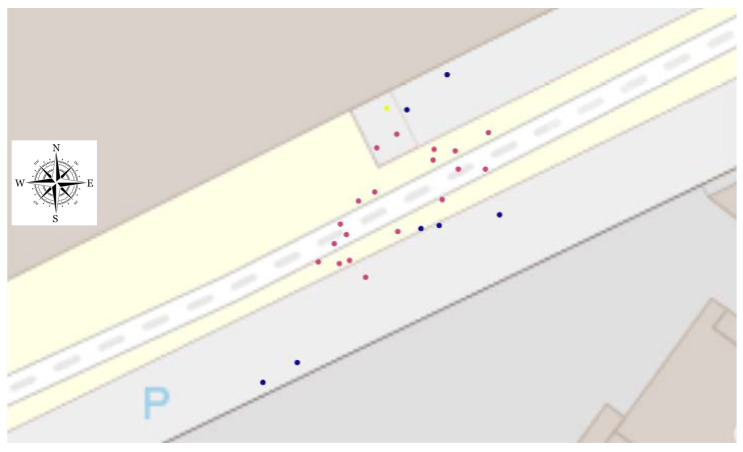
Positioning of users on a cadastral map (yellow: camera position, blue: cars and red: pedestrians).

As can be seen in Figure 17, the location of the camera mast (the yellow dot) appears slightly offset (less than a meter) in the northeast direction. The main sources of error are related to the uncertainties concerning, on the one hand, the non-verticality of the mast on which the camera is attached and, on the other hand, the azimuth angle of the optical axis of the camera. Indeed, the plumb of the mast supporting the camera was not checked when it was erected, and the azimuth of the camera was measured using a simple magnetic compass. In both cases, an error of a few degrees generates positioning errors of several tens of centimeters. A study of the sensitivity to these parameters is planned.

## 5. Discussion

The results shown in Section 4 demonstrate that our system is able to properly detect, classify, and localize road users in a GNSS coordinate system in real time, even in low-light-illumination conditions.

The supervisor transmits a list (*φ_u_*, *ω_u_*, *Label_u_*) of all GNSS positions and labels to vehicles present in the area. A particular vehicle is able to recognize itself by GNSS coordinates matching among the transmitted list. Its embedded GNSS is able to position the other users on the navigation screen and warn the driver in the case of imminent danger.

The error of the localization is in the order of a meter. The velocity estimation is performed offline, and the error is below one meter per second for urban speed range (0 to 15 m/s). Moreover, thanks to the almost ortho-photographic configuration of the imaging system, the anonymity of all street users is guaranteed.

Our system does not require a tracking algorithm and, thus, it is not too sensitive on a few missed detections.

The quality of the localization of the detected objects is strongly influenced by the shooting parameters (*H*, *f*, *α*, and *γ*), which requires a precise accelerometer and magnetometer. An alternate solution would be to use the GNSS position of a particular point on the ground in order to auto-calibrate the camera to world transform.

**Figure 18:**
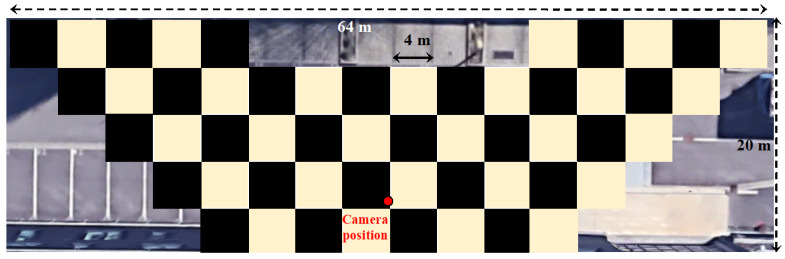
Effective horizontal area in the dewarped image.

Each use case requires the choice of an appropriate optical configuration, in particular for the parameters *H* and *α*. In the experiment described in Section 4, the tilt of the camera favors the frontal zone over a depth of 20 m and a median width of about 50 m. The effective observation area is highlighted on the dewarped aerial view in Figure 18. This configuration is well suited to standard urban street surveillance (road + sidewalk).

Our reflex camera uses a 16:9 format in video mode. A 4:3 format or, even better, 1:1 (very rare) would allow for a better exploitation of the optical field of view.

The height of the objects can introduce a positioning bias. For low-cost lens usage, a complete tabulation of the distortion is necessary.

## 6. Conclusions and Future Work

We propose a system that is able to properly detect, classify, and localize road users in a GNSS coordinate system in real time, even in low-light-illumination conditions. Very few works carried out in the same conditions have been published. Papers addressing the fisheye camera problem for detection have been previously published [20,21]. However, to the best of our knowledge, no one has simultaneously tackled low-light-illumination conditions and quasi ortho-photographic shooting.

Improvements are required to retrain the detector with a larger set of quasi ortho-photographic images, on the one hand, and to integrate a form of semantic segmentation in order to be able to transmit additional information (for example, “pedestrian on the roadway”) on the other hand. Concerning the real-time processing aspects, we seek to replace the computation of the optical flow with a neural network by another approach that is less greedy in computing resources to obtain a real-time demonstrator implemented on an embedded GPU platform.

## Figures and Tables

**Table 1:** Comparison between dimensional measurements on the ground and estimation using the system.

	Ground Truth	Estimation on Fisheye View	Relative Difference		Ground Truth	Estimation on Fisheye View	Relative Difference
*d*_1_	4.8	4.7	−2.1%	*d*_5_	19.5	19.5	0%
*d*_2_	4.7	4.5	−4.3%	*d*_6_	10	11	10%
*d*_3_	4.8	5.2	8.3%	*d*_7_	1.8	1.8	0%
*d*_4_	1.5	1.4	−6.7%	*d*_8_	1.5	1.5	0%

## Data Availability

Not applicable.
